# Comparison between the results of simulated mechanical imaging on software breast phantom and *in vivo* measurements

**DOI:** 10.1093/rpd/ncaf178

**Published:** 2026-03-13

**Authors:** John-Henry Markbo, Predrag R Bakic, Hanna Isaksson, Kristin Johnson, Magnus Dustler

**Affiliations:** Translational medicine, Lund University, Jan Waldenströms gata 35, 214 28, Malmö, Sweden; Translational medicine, Lund University, Jan Waldenströms gata 35, 214 28, Malmö, Sweden; Radiology, University of Pennsylvania, 3451 Walnut Street, Philadelphia, PA 19104, United States; Biomedical Engineering, Lund University, Sölvegatan 19, 223 62, Lund, Sweden; Translational medicine, Lund University, Jan Waldenströms gata 35, 214 28, Malmö, Sweden; Department of Imaging and Physiology, Skåne University Hospital, Inga Marie Nilssons gata 47, 214 28, Malmö, Sweden; Translational medicine, Lund University, Jan Waldenströms gata 35, 214 28, Malmö, Sweden

## Abstract

The focus of this study was to evaluate the feasibility of incorporating Perlin noise, a method that has previously been used to model the tissue distribution of software breast phantoms, when creating a ‘compressible’ software breast phantom used in finite element analysis. Several compressible phantoms were created to represent different stages of aging in a virtual patient when younger, fibroglandular tissue is more prevalent than adipose tissue. During the ageing process, this ratio changes so that the breast contains more adipose tissue and less fibroglandular tissue. When simulating the compression of these phantoms, the determined reaction forces on the simulated compression plate increase with higher breast density. The resulting reaction forces on the compression plates are well above the values from *in vivo* measurements performed by our research group. However, when considering the differences between the procedures in the two studies, the simulated results are arguably comparable to the *in vivo* measurements.

## Introduction

Breast cancer is the most common form of cancer among women globally today [1]. Breast cancer screening enables early detection, which improves outcomes through earlier treatment [2]. As such, we need to continuously assess current cancer detection methods, as well as develop novel methods, to be able to further improve the cancer detection in screening for earlier detection and, in effect, more successful treatment.

Traditionally, this assessment is made through clinical trials of medical imaging systems for breast cancer screening. An example of such a system is Digital Mammography (DM). As with many diagnostic procedures, performing DM trials is expensive and time consuming. Also, the risk of developing cancer increases with exposure to ionizing radiation, and therefore, repeated imaging of the same patient is rarely motivated [3]. Virtual Clinical Trials (VCTs), an umbrella term referring to the simulation of clinical interventions such as imaging, and results thereof, offer an affordable and time-efficient method of emulating the clinical process and can produce results of virtual patients repeatedly.

The use of VCTs is only feasible if the simulated material is realistic enough for the specified task. To achieve this, our lab has pioneered the use of Perlin noise [4, 5] to create 3D software breast phantoms, which are made to replicate the distribution and composition of tissue in a breast. First developed in 1985, Perlin noise is often used in computer graphics and video game development to replicate the appearance of natural or organic structures, such as clouds or waves on water. It has also been used before in previous studies, such as by Tomic *et al.* [6, 7] and Dustler *et al.* [8, 9] for the simulation of breast tissue in DM.

A novel breast cancer imaging method that our team has been developing is Mechanical Imaging (MI). This method visualizes the distribution of the surface stress of the breast during the mammographic process. This visualization is yielded from a grid of pressure sensors on the DM compression paddle, providing information about local elastic properties of the tissue in the compressed breast. This can be seen as an extension of classical detection through palpation performed by a medical practitioner.

The pressure measurements from MI will depend on the composition of tissue in the breast. Breast density is a term used to describe the ratio of fibroglandular tissue and adipose tissue, where a high ratio of fibroglandular tissue equals high density. Also, as the ratio of fibroglandular tissue increases or decreases, so will the overall stiffness of the breast change and, in effect, it is assumed, so will the pressure measurements obtained through MI.

MI has been shown by Dustler *et al*. [10] to reduce false positives in breast cancer screening when used in conjunction with clinical mammography. As the use of Perlin noise has shown promise before in VCTs for DM, it raises the question of whether Perlin noise could also be used to model a software breast phantom that is subjected to compression. openVCT [11], a VCT software, simulates the compression of a software breast phantom and the resulting displacement of tissue, but that software breast phantom is assumed to have a homogenous structure, which makes it unsuitable for simulating the detailed distribution of anatomy-dependent surface stress needed for MI. For this reason, we are investigating the feasibility of incorporating Perlin noise to model the heterogeneous structure of breast tissue during compression.

The purpose of this study is to evaluate how differences in density of a Perlin noise-based software breast phantom affect the results of simulated MI, and if our virtual compressible (Perlin noise−based) breast phantom yields results that are close to observed clinical results.

## Materials and Methods

### Mechanical imaging

**Figure 1:**
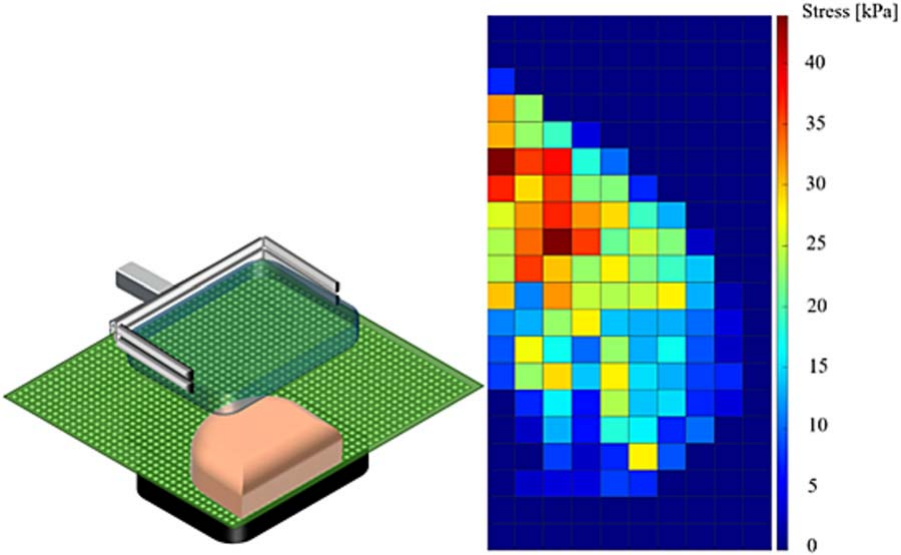
Schematic image (left) of using the TekScan sensor during the mammographic process. A breast can be seen placed on the sensor, both of which are positioned between two compression paddles. An example of clinical measurement of MI using this setup can be seen on the right, with stress distribution in kPa.

MI measurements are performed using a pressure sensor (TekScan) placed against the stationary mammographic paddle. The spatial resolution of the sensor is 1 cm × 1 cm. In Fig. 1, a schematic image of the setup and an example of a measurement of a clinical MI examination can be seen.

### Perlin noise−based software breast phantom

**Figure 2:**
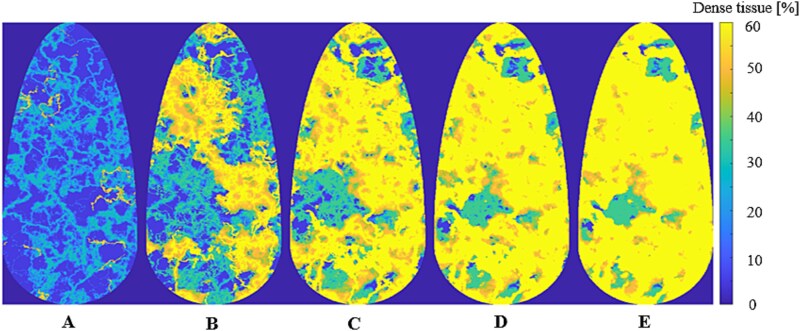
2D representations of the software phantoms A, B, C, D, and E used in this study. These phantoms have different amounts of dense tissue corresponding to alphabetical order: A having the least amount and E having the highest amount. The amount of dense tissue in each image pixel is represented by the color explained by the colorbar on the right in terms of percentage.

Perlin noise is a method of creating coherent noise without discontinuities. The resulting appearance yielded by Perlin noise resembles organic and natural structures. The noise is modelled on a grid over an area by assigning (at random) vector values from a unit cube node points to the intersection points of the grid. For any arbitrary point on the grid, the noise value is calculated from the scalar products of the distance vectors to each of the values of the intersection points it is enclosed by. This means that the value of the Perlin noise function at the grid points is always zero. Using this, we can control the size and variability of the Perlin noise by changing the layout of the grid; a grid with a large distance between the intersection points will create larger structures with lesser variability, while smaller distances will create small structures of large variability. Using different grid points is referred to as using octaves (which is a term from musical theory to denote a doubling of the value of frequency). So, using a grid that is an octave ‘higher’ means that the distance between intersection points is halved, while the distance is doubled for a ‘lower’ octave. The octaves are used as base functions that are superimposed on each other to create a representation of realistic-looking structures. We performed simulations of MI using software breast phantoms with different densities. The tissue composition of the phantom was based on an in-house-developed Perlin model of breast anatomy. The method can be used to model progressive changes in the amount of dense tissue within a certain simulated breast, to model the diminishing of the amount of fibroglandular tissue in a breast, which is part of the natural aging process [12]. To simulate the aging process, the values making up the Perlin noise structure were thresholded. The thresholding parameters are chosen so that more of the Perlin noise structure is made of adipose values when simulating older breast tissue, and for younger breasts, the parameters are chosen so as to let more of the structure be made up of fibroglandular tissue. The different types of phantoms are labelled from A-E, where the phantoms A, B, C, D, and E contain 9%, 27%, 43%, 53%, and 56% dense tissue, respectively (Table 1). A visual example of this gradual change in the software breast phantom is presented in Fig. 2.

**Table 1:** Overview of the simulated results when using breast phantoms of different densities, here labeled from A to E in order of least amount of dense tissue, to the most amount of dense tissue.

Density label	A	B	C	D	E
Dense tissue [%]	9	27	43	53	56
Reaction force [N]	65	92	115	129	133
Contact area [cm^2^]	49	49	49	49	49
Mean stress [kPa]	13	19	23	26	27

The voxel values in our anatomical breast model have discrete indexes corresponding to a preset table of ratios between adipose and fibroglandular tissue. These voxel values range from 1 to 0.40; the value of 1 representing a tissue mixture with 100% adipose tissue, and a value of 0.40 representing a tissue mixture of 40% adipose tissue and 60% fibroglandular tissue (representing the highest value visualized in Fig. 2), similarly to the Perlin noise−based software breast phantoms described by Tomic *et al*. [5]. The difference between the breast models is the number of voxels that have a higher ratio of adipose tissue, i.e. more voxel values closer to the value of *1*. The reduction of tissue is modeled by thresholding the Perlin noise structure at different values to change the rate of adipose tissue, i.e. simply increasing the number of voxels with values closer to *1* to simulate the aging process.

### Finite element analysis

The MI simulations were performed by finite element analysis (FEA) using the software FEBio [13]. The shape of the deformable breast phantom was created by connecting two quarters of two different ellipsoids, forming a breast-like shape (roughly made to correspond to cup size A) with dimensions spanning 17 cm × 7.5 cm × 3.7 cm. The software phantom was partitioned into 122 556 tetrahedral elements in an FEA mesh using iso2mesh software in Matlab. To replicate the hyperelastic properties of breast tissue, a neo-Hookean material model was used. The values of the stiffness modulus were assigned according to the range given by Krouskop *et al*. [14] for breast tissue. For our simulations, we used the stiffness modulus of 20 kPa for adipose tissue and 80 kPa for fibroglandular tissue.

The compression paddles are modeled with plates that were composed of 60 000 hexahedral elements with 400 × 150 elements in the *x*- and *z*-directions for a total of 20 cm × 1 cm × 7.5 cm. The back of the breast phantom was kept fixed in the *z*-direction, so as to not permit tissue to be simulated as being pushed into the chest wall. The phantom surface nodes that were within 8 mm of distance of the plate (along the *y*-axis) were fixed in the *x*-direction, with one phantom node in contact with the nonmoving compression plate being fixed in all spatial directions.

**Figure 3:**
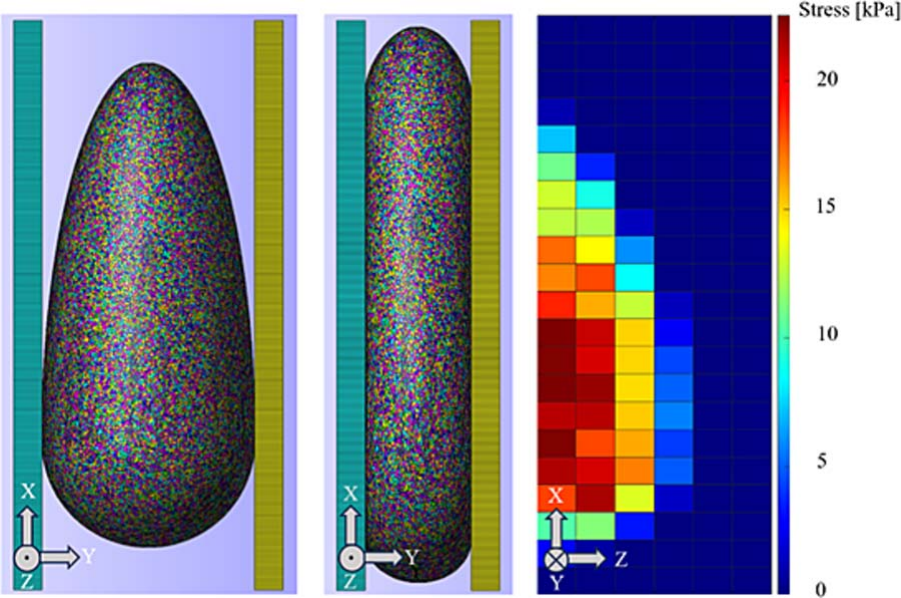
Example of software breast phantom before compression (left), and after compression (middle). The simulated MI response (right) yields the simulated pressure in patches of 1 cm^2^ each [kPa]. The *x*-axis corresponds to the craniocaudal direction, the *y*-axis corresponds to the mediolateral direction, and the *z*-axis corresponds to the ventrodorsal direction.

The contact between the compression plates and the phantom mesh was set to ‘facet-to-facet sliding’. One of the compression plates was fixed in all directions of space, while the other was only fixed in the *x*- and *z*-directions and prescribed a movement along the *y*-axis corresponding to a change of position by 3.76 cm, which is about half the phantom’s width (in the *y*-direction). The visualization of this compression can be seen in Fig. 3. The plates were assigned a high modulus of 200 kPa, to account for their rigidity in comparison to the breast tissue. The forces in the direction of compression were obtained at each node of the plates. The total mean stress was calculated from the total force (in the *y*-direction) divided by the total area of the contact surface between plate and phantom. The simulated MI (Fig. 3) was calculated in the same manner, by extracting the force at the nodes on the compression plate but instead summing up the forces in 1 cm × 1 cm patches on the plate and dividing by that patch area.

The elements in the FEA mesh were assigned a stiffness modulus value corresponding to the weighted average of the values of the voxels of the Perlin noise–based software breast phantom. For each tetrahedron, the voxels that had a position corresponding to the inside of the FEA element were found, and the weighted average was found using numerical integration.

## Results

The results for compression of the five software phantoms are shown in Table 1. The determined reaction forces on the plates were, in order of increasing amount of dense tissue present in the software phantoms: 65, 92, 115, 129, and 133 N. The contact surface areas after compression were all 49 cm^2^ for all breast phantoms, independent of the ratio of dense tissue within the phantoms. The corresponding total mean pressure to the five software phantoms is thus the following: 13, 19, 23, 26, and 27 kPa.

## Discussion

The simulated measurements of mean stress on the contact area are well above the range of mean values reported by a previous *in vivo* study by our research group [15]. This study reports an average of mean stress over different MI measurements to be 2.5 kPa using a rigid plate for compression in the craniocaudal view. This is well below the lowest value of average pressure on the phantoms in this study, which was determined to be 13 kPa (Table 1). The *in vivo* study used an applied force of ~100 N on the plates, which is within the range of the reaction forces calculated on the simulated plates in this study, which spanned 65−133 N (Table 1). So even though there is comparable force involved on the plates, the *in vivo* pressure measurements of this previous study are quite a bit lower than the results presented in this study. However, the *in vivo* measurement study also reports findings that might account for this discrepancy: Only a fraction, on average 27% (for the craniocaudal view), of the applied force on the plates is distributed over the central breast area. This is theorized to be because of the chest wall area (including the pectoral muscles) absorbing a large share of the applied compressive force. The higher values of simulated pressures in this study might be explained by the simulation process: we are simulating the compression of a detached breast, so to speak, and the force resulting from the compression will only be able to distribute over that confined breast volume. This is quite a different setup from the *in vivo* measurements with which we are comparing our simulated results, where forces applied on the breast may propagate into adjacent body structures (i.e. the chest wall).

As it stands, the range of our simulated results of pressure are in the order of one magnitude higher than the results from the *in vivo* measurements; but by accounting for the redistribution of force away from the breast area observed in the *in vivo* study, only retaining 27% of the value of reaction forces determined in the simulations, the resulting average pressures from our simulations does then come within the same order of magnitude as the average value of 2.5 kPa (craniocaudal view) found in the *in vivo* measurements. Furthermore, the values of average stress being reported are calculated using the total ‘projected’ breast area, not the area in contact with the plates. This projected area will always be larger than (or possibly equal to) the breast area that is in actual contact with the compression plates. Using projected area instead of contact area is likely to have caused the reported results to be lower than if contact area had been used in the calculations. This is because pressure is inversely proportional to area for a given force. Additionally, the *in vivo* measurements are based on a specific, applied force causing the compression. The simulated results, on the other hand, were instead yielded by a specific displacement of the compression plates. For future studies, the simulations should be changed appropriately to mimic the clinical procedure of mammographic compression better.

For future studies, we plan to analyze the average stress directly from clinical data when comparing the results of simulated and clinical MI, instead of comparing MI results from the literature, to account for differences in methods of calculating the average values of MI measurements. We are not currently simulating the presence of ligaments or pectoral muscle, which are tissue types with relatively high stiffness moduli. Implementing a software breast phantom with these tissues present would therefore likely cause even higher values in stress when simulating MI. Another reason that could explain the higher stress values in the simulated results is the lack of a chest wall in the model, which, if present, could absorb a large part of the applied force due to displacement of the mammographic paddle, as in the clinical experiments. Finally, the values reported by Krouskop *et al*. [12], for the stiffness moduli concerning the different kinds of tissue, vary widely: from 9 to 31 kPa for adipose tissue, 14 to 83 kPa for glandular tissue, and 62 to 329 kPa for fibrous tissue. In future studies, this variation should be taken more into account to cover the possible spectrum of MI results that can be yielded from using the full range of possible combinations of stiffness moduli for different breast tissues.

## Conclusion

In this study, software breast phantoms were created using the Perlin noise, where the structures contain differing degrees of density (of fibroglandular tissue), but with the same underlying tissue structure. We have shown that the reaction force on the simulated compression plates does increase with a higher amount of fibroglandular tissue.

There are differences in procedure between the simulations in this study and the *in vivo* measurements that should be considered, and mitigated, for future studies. However, by accounting for those differences between *in vivo* measurements and the simulations in this study, the simulated results are deemed feasible.
